# Suppression of *CINNAMOYL*-*CoA REDUCTASE* increases the level of monolignol ferulates incorporated into maize lignins

**DOI:** 10.1186/s13068-017-0793-1

**Published:** 2017-05-02

**Authors:** Rebecca A. Smith, Cynthia L. Cass, Mona Mazaheri, Rajandeep S. Sekhon, Marlies Heckwolf, Heidi Kaeppler, Natalia de Leon, Shawn D. Mansfield, Shawn M. Kaeppler, John C. Sedbrook, Steven D. Karlen, John Ralph

**Affiliations:** 1 Department of Energy Great Lakes Bioenergy Research Center, The Wisconsin Energy Institute, University of Wisconsin-Madison, 1552 University Avenue, Madison, WI 53726-4084 USA; 2 Department of Biochemistry, University of Wisconsin-Madison, Madison, WI 53706 USA; 3 Department of Energy Great Lakes Bioenergy Research Center, School of Biological Sciences, Illinois State University, Normal, IL 61790 USA; 4 Department of Agronomy, University of Wisconsin-Madison, Madison, WI 53706 USA; 5 Department of Wood Science, University of British Columbia, Vancouver, BC Canada; 6 Department of Genetics and Biochemistry, Clemson University, Clemson, USA

**Keywords:** *Zea mays*, Mass spectrometry, Cell wall digestibility, Biofuels

## Abstract

**Background:**

The cell wall polymer lignin provides structural support and rigidity to plant cell walls, and therefore to the plant body. However, the recalcitrance associated with lignin impedes the extraction of polysaccharides from the cell wall to make plant-based biofuels and biomaterials. The cell wall digestibility can be improved by introducing labile ester bonds into the lignin backbone that can be easily broken under mild base treatment at room temperature. The FERULOYL-CoA MONOLIGNOL TRANSFERASE (FMT) enzyme, which may be naturally found in many plants, uses feruloyl-CoA and monolignols to synthesize the ester-linked monolignol ferulate conjugates. A mutation in the first lignin-specific biosynthetic enzyme, CINNAMOYL-CoA REDUCTASE (CCR), results in an increase in the intracellular pool of feruloyl-CoA.

**Results:**

Maize (*Zea mays*) has a native putative FMT enzyme, and its *ccr* mutants produce an increased pool of feruloyl-CoA that can be used for conversion to monolignol ferulate conjugates. The decreased lignin content and monomers did not, however, impact the plant growth or biomass. The increase in monolignol conjugates correlated with an improvement in the digestibility of maize stem rind tissue.

**Conclusions:**

Together, increased monolignol ferulates and improved digestibility in *ccr1* mutant plants suggests that they may be superior biofuel crops.

**Electronic supplementary material:**

The online version of this article (doi:10.1186/s13068-017-0793-1) contains supplementary material, which is available to authorized users.

## Background

One of the front contenders to replace the currently used petroleum fuel sources is cellulosic biofuels. Plant material, such as corn stover, is an excellent source of cell wall material, but the lignin in the wall makes it difficult to deconstruct and effectively recover the valuable polysaccharides for conversion to fuels and other coproducts. Lignin is a complex phenolic polymer built from a variety of phenolic monomers coupled together with an assortment of C–C and C–O–C (ether) linkages. As a result, the absolute structure of lignin is not defined, and the polymer cannot be easily degraded because of the difficulty in cleaving its C–C and even its ether bonds. Studies are therefore underway to reduce the recalcitrance of lignin without negatively impacting the growth and development of the plant.

Recent success in this field was achieved by introducing an exotic *FERULOYL*-*CoA MONOLIGNOL TRANSFERASE* (*FMT*) gene from *Angelica sinensis* into poplar (*Populus alba* × *grandidentata*) [1] resulting in trees that produce an FMT enzyme responsible for synthesizing monolignol ferulate conjugates. Monolignol ferulates (ML-FAs) are derived from FMT-catalyzed acylation of lignin monomeric units, monolignols (ML), at the γ-hydroxy position by ferulate (FA), via feruloyl-CoA, an intermediate in the lignin biosynthetic pathway. The result was poplar trees with higher levels of ML-FAs incorporated into the lignin. Unlike the least recalcitrant of the interunit linkages in the lignin polymer, the β-ethers that require ~130 °C to cleave at a moderate rate of 10^−3^/min in 1 M NaOH [2], the esters in these modified lignins were easily broken by base treatment even at room temperature, improving the saccharification of the wood [1]. As the trees did not experience any significant growth defects, this study suggested that the monolignol ferulate strategy had enormous potential for the development of plants for biofuel and biomaterials applications.

Recent improvements in the detection sensitivity for releasable diagnostic ML-FA-derived components have led to the discovery that wild-type poplar also naturally produces ML-FAs, albeit at a lower level, and incorporates them into the lignin polymer. Therefore, poplar must have a native FMT enzyme, or a multi-functional enzyme with FMT capabilities. Other major bioenergy crops have also been shown to have natural levels of ML-FAs incorporated into their lignins [3]. These include many economically important monocot and eudicot plant species, such as maize, switchgrass, sorghum, miscanthus, and eucalyptus.

**Fig. 1:**
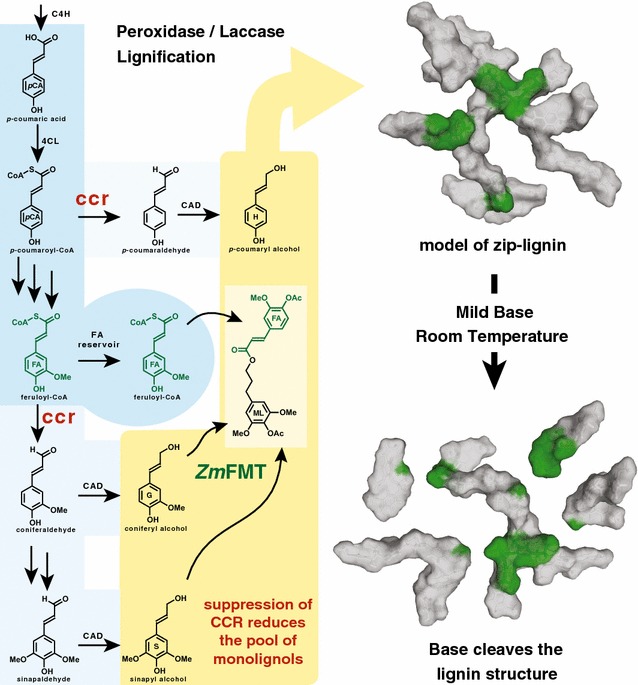
An abbreviated version of the lignin biosynthetic pathway highlighting the effects of the *ccr1* mutation. The mutated *CCR1* gene results in reduced *CCR1* transcript, and presumably less CCR1 enzyme, and a pool of feruloyl-CoA (FA reservoir). The conjugates formed between the monolignols and feruloyl-CoA by *Zm*FMT are incorporated into the lignin polymer, generating a zip-lignin in which the ester bonds in the backbone are readily cleaved by base

Given that ML-FAs, and therefore presumably FMT enzymes, are naturally occurring, we hypothesized that the levels of ML-FAs could be manipulated by perturbing the relative levels of the substrate pool available to the FMT enzyme. Higher levels of ML-FAs could prove valuable, as there is a correlation between the levels of ML-FAs and the digestibility of the cell wall following alkaline pretreatments [1, 4]. Previous studies of the lignin biosynthetic pathway have shown that downregulating the *CINNAMOYL*-*CoA REDUCTASE* (*CCR1*) gene, which encodes an enzyme with the main function of converting feruloyl-CoA into coniferaldehyde, results in a pool of feruloyl-CoA (FA-CoA) and its derivatives [5–7]. The levels of ML-FAs in lignin biosynthetic mutants, such as *ccr1* mutants, have not previously been investigated. Increasing the pool of the FA-CoA intermediate could therefore provide more substrate for the naturally occurring FMT enzyme and increase the amount of ML-FAs, which could in turn improve the digestibility of the cell walls, as outlined in Fig. 1.

To test this hypothesis, we examined the levels of ML-FAs releasable from a maize *ccr1* mutant line. Maize is a biofuel crop of interest as well as a valuable model monocot system. As we illustrate, maize *ccr1* mutant plants, hypothesized to have an increased pool of the feruloyl-CoA substrate, displayed markedly higher levels of lignin-incorporated ML-FAs, along with lower total lignin, and improved biomass digestibility, without detriment to overall plant growth.

## Results and discussion

**Fig. 2:**
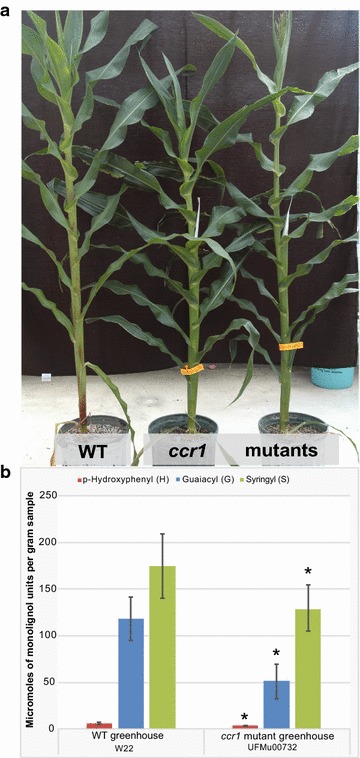
Biomass and lignin monomer composition data from wild-type and *Zmccr1* mutant plants. **a** The *Zmccr1* mutant plants appear no different from wild type in growth and biomass yield when grown in the greenhouse. **b** The levels of *p*-hydroxyphenyl (H), guaiacyl (G), and syringyl (S) monomers released by thioacidolysis are all significantly lower in the *Zmccr1* mutants relative to those in wild-type (WT) plants. *Error bars* represent standard deviations of biological replicates (*n* = 5 plants), *asterisks* represent a significant difference from WT, *p* < 0.05

To examine the levels of ML-FAs incorporated into *ccr1* mutants, we used established genetically uniform W22 maize inbred lines obtained from the UniformMu collection [8]. The line analyzed in this study harbors a Mu transposon in exon four of the *CCR1* gene (GRMZM2G131205, Additional file 1: Fig. 1A, B); for further details, see Materials and Methods. Mutant *ccr1* maize plants (*Zmccr1*) and wild-type W22 controls were grown in both the greenhouse and the field, and stem tissue from all plants were sampled at maturity. Reverse-transcription semi-quantitative PCR determined that plants homozygous for the mu1013391 insertion had reduced *ccr1* transcript present compared to wild type (Additional file 1: Fig. 1C). A complete knock-out of the *CCR1* gene has been reported to cause severe growth defects and drastic decreases in total lignin content in various monocots and eudicots [5, 7, 9, 10]. Despite the reduced *ccr1* transcript level, the maize plants here appear no different from wild type in their growth and biomass yields when grown in a greenhouse (Fig. 2a). This has previously been reported for other *ccr* mutants in maize, which had normal seed germination, growth, and development [11].

The total lignin level and lignin monomer content were analyzed for each line by Klason lignin analysis and thioacidolysis, respectively. The acid-insoluble lignin content was significantly decreased in the *ccr1* plants relative to wild type (17.7 versus 22%; Table 1). Surprisingly, this lignin decrease did not negatively impact the growth of the mutant plants. Correlated with the lower lignin level, the total amount of released guaiacyl (G) and syringyl (S) thioacidolysis monomers was also lower, as has been previously reported [7, 9]. Thioacidolysis releases monomers by selectively cleaving β–O–4-linkages between monomeric units; the monomer yields therefore represent only a fraction of the units (~30%), but reflect the approximate proportion of each monolignol incorporated into the lignin polymer (see Table 2 for calculation of theoretical yields). As anticipated for a mutation in the first lignin-specific enzyme in the phenylpropanoid pathway, the *ccr1* mutation reduces all monolignol biosynthesis. The major difference was observed in G monomers (30–71 micromoles per g AIR in mutants versus 100–133 in WT), as has been noted previously when the flux into the monolignol pathway is reduced (Fig. 2; Table 1); the *p*-hydroxyphenyl (H) and S monomers were also lower in the mutant (approximately 40 and 30% decrease, respectively).

**Table 1:** Chemical analysis values for wild-type (WT) and *ccr1* mutant plants

	UFMu00732 *ccr1* mutants (greenhouse)	WT W22 maize plants (greenhouse)	UFMu00732 *ccr1* mutants (field-grown)	WT W22 maize plants (field-grown)
% Acid-insoluble Klason lignin	17.7% ± 0.8	22.0% ± 0.1	17.3% ± 0.2	22.8% ± 0.2
Thioacidolysis				
S:G:H ratios	71:27:1.6	59:40:1.7	68:28:4	60:37:3
H (μmol/g)	2.9 ± 0.4	4.9 ± 1.1	2.8 ± 0.1	4.2 ± 0.2
G (μmol/g)	50.6 ± 18.4	117.1 ± 23.0	18.9 ± 1.3	48.1 ± 0.74
S (μmol/g)	127.8 ± 25	173.9 ± 34.2	45.5 ± 2.3	78.1 ± 2.0
DFRC				
CA-DHFA nmol/g lignin	96.4 ± 30.7	107.6 ± 31.0	95.9 ± 8.2	123.4 ± 19.3
SA-DHFA nmol/g lignin	911.7 ± 141.1	308.9 ± 3.2	1775.7 ± 39.0	1039.4 ± 139.9
CA-DH*p*CA nmol/g lignin	12,554.9 ± 2453.6	9551.8 ± 1220.8	13,739.8 ± 18.0	12,730.1 ± 621.5
SA-DH*p*CA nmol/g lignin	319,314.6 ± 33,334.7	275,157.7 ± 850.4	238,058.8 ± 4112.7	292,782.2 ± 20,356.2
Digestibility				
% Glucose	17.9% ± 1.8	11.7% ± 1.4	16.0% ± 0.1	11.4% ± 0.3
% Pentose	6.4% ± 0.7	4.3% ± 0.6	4.9% ± 0.2	4.2% ± 0.3

**Table 2:** Calculation of the percentage of lignin quantified by DFRC and thioacidolysis

Chemical assay	DFRC	Thioacidolysis
Whole cell-wall sample	50 mg	50 mg
Klason lignin (wt%)	22%	22%
Approximate mass of lignin (wt% Klason lignin × 50 mg wcw)	11,000 µg	11,000 µg
Mass of products quantified (µmol/mg wcw × µg/µmol)	2300 µg	2900 µg
Weight% of lignin detected	21%	26%

Component compositional analysis of lignin by degradative assays, thioacidolysis, and DFRC, only accounts for a portion of the total lignin structure. A calculation approximating the amount of lignin that each of these assays measures can be performed by comparing the weight of the components that are quantified and the amount of Klason lignin in the sample. In this study, wild-type maize rind tissue was ~22% Klason lignin. The detected product was then converted to the weight of the lignin components, i.e., the H, G, S, ML-DH*p*CA, and ML-DHFA structures are converted into the non-functionalized monolignols and hydroxycinnamic acids. Taking the summation of the weight of these components and dividing mass of lignin gave an approximate weight% of the lignin that was detected. The products of the DFRC analysis of wild-type maize rind tissue in this study accounted for ~21% of the total lignin, whereas the product mix from thioacidolysis accounted for ~26% of the lignin

**Fig. 3:**
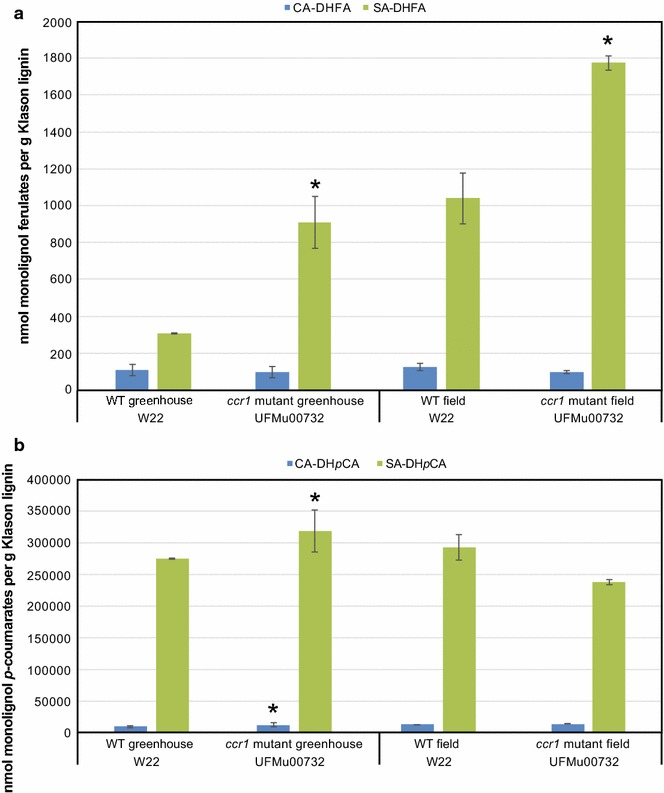
The levels of monolignol conjugates released from DFRC analysis. **a** The level of monolignol dihydroferulate (ML-DHFA) conjugates released from *Zmccr1* mutants is three to fivefold higher than from wild-type (WT) plants, indicating higher ML-DHFA production and incorporation into the lignin polymer. **b** The level of monolignol dihydro-*p*-coumarates (ML-DH*p*CA) is similar between the *Zmccr1* mutants and the wild-type plants, indicating that the production of monolignol ferulates and *p*-coumarates are independent. CA-FA/*p*CA is the coniferyl alcohol conjugate, SA-FA/*p*CA the sinapyl alcohol conjugate. *Error bars* represent standard deviation of biological replicates (*n* = 5 plants for greenhouse lines, *n* = 2 pools of 10 plants for field-grown lines), *asterisks* represent a significant difference from WT, *p* < 0.05

The levels of monolignol conjugates in the lignin polymer, and not just monolignol levels, were of particular interest in the *ccr1* mutant plants because of our hypothesis that the increased pool of feruloyl-CoA generated by reduced CCR1 activity could lead to increased production of ML-FAs. Derivatization Followed by Reductive Cleavage (DFRC), a method that cleaves β-ethers but leaves γ-esters intact, was the first method to effectively examine and relatively quantify the incorporation of ML-FAs into the lignin polymer [1]. The method liberates diagnostic ester conjugates but, due to the complex and combinatorial manner that ML-FAs incorporate into lignins (see Fig. S2 in [1]), only a small fraction is released and quantifiable. DFRC analysis of maize wild-type (W22 background) and *ccr1* mutant plants revealed a three- to fivefold higher level of ML-FAs released from *ccr1* relative to wild-type plants (300 versus 800–1200 nmol ML-FA per gram lignin). The elevated level was the result of higher quantities of both coniferyl and sinapyl ferulates, but sinapyl ferulates are much more prominent (Fig. 3, Additional file 1: Fig. 2), as is typical for most monocot species [3]. This result indicates that the pool of feruloyl-CoA generated by the *ccr1* mutation is used by the putative natural maize *Zm*FMT to generate more monolignol conjugates that are then used for lignification.

In addition to monolignol ferulate conjugates, maize also naturally produces another class of conjugates, the monolignol *p*-coumarates (ML-*p*CA) via the *p*-COUMAROYL-CoA MONOLIGNOL TRANSFERASE (*Zm*PMT, also referred to as *p*CAT) enzyme [12]. PMT synthesizes ML-*p*CAs by the conjugation of monolignols to *p*-coumaroyl-CoA [13, 14]. The levels of ML-*p*CAs were not significantly altered in the *ccr1* mutant plants that had the higher ML-FA quantities relative to wild-type plants (Fig. 3). This indicates that the production of ML-FAs and ML-*p*CAs are independent, and are most likely synthesized via separate enzymes, i.e., the putative *Zm*FMTs and *Zm*PMTs, respectively.

As a negative control to ensure that the increased pool of feruloyl-CoA was correlated with the increased monolignol ferulate levels (i.e., that natural FMTs are using (some of) the excess feruloyl-CoA to generate ML-FAs), *Arabidopsis thaliana ccr1* knock-out mutants were examined for the presence of ML-FAs. Arabidopsis does not have an FMT enzyme, as discerned from the absence of ML-FAs, or indeed any of the monolignol conjugates, in the wild-type plants [3]. The Arabidopsis *ccr1* mutants did not produce any detectable level of ML-FAs (Additional file 1: Fig. 2), but did generate a pool of ferulate derivatives [5], supporting the idea that the increase in ML-FAs in the maize mutants is the result of FMT activity (that is absent in *Arabidopsis*).

**Fig. 4:**
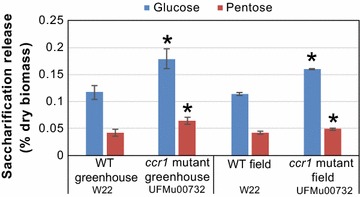
Relative glucose and pentose release from limited-extent digestibility of *Zmccr1* mutant lines is higher than from wild-type (WT) plants, both in greenhouse and field-grown maize. *Error bars* represent standard deviations of biological replicates (*n* = 5 plants for greenhouse lines, *n* = 2 pools of 10 plants for field-grown lines), *asterisks* represent significant difference from WT, *p* < 0.05

In FMT-poplar trees, the increased production of ML-FAs was correlated with increased cell wall digestibility following mild base pretreatment [1]. We subjected the maize *ccr1* mutant plants, with their higher levels of ML-FAs, to partial saccharification analysis, following mild base (6.25 mM NaOH, 90 °C for 3 h) pretreatment. The digestibility of the *ccr1* senesced tissues, as measured by glucose release, was significantly higher than that of wild-type plants (~15 to 20 versus 10%, respectively; Fig. 4; Table 1). This improvement may be due, in part, to the decreased total lignin level in the plants but, with the precedent set by Wilkerson et al. [1] of the correlation between ML-FA levels and improved digestibility in plants without changes in total lignin, is also likely related to the increased levels of ML-FAs incorporated into the lignin. Park et al. [11] reported improved digestibility of *RNAi*-*CCR1* maize plants when pretreated by ammonia fiber expansion (AFEX).

Taken together, the growth and chemical analysis data all suggest that greenhouse-grown *ccr1* mutants have excellent potential for biofuels production. Greenhouse conditions, however, are optimal for plant growth and are not necessarily representative of how the plants perform in the field. Field trials of *ccr1* mutants demonstrated that the *ccr1* mutation does not confer any deleterious effect on agronomic performance and, in fact, the *ccr1* mutant plants produced significantly more biomass than wild-type control maize plants (Table 3). The Klason acid-insoluble lignin levels matched those of the greenhouse-grown wild-type and *ccr1* mutant plants (Table 1). The field-grown wild-type stems had levels of ML-FAs similar to those of greenhouse-grown *ccr1* mutants, whereas the field-grown *ccr* plants had an almost twofold higher ML-FA release than greenhouse-grown *ccr1* mutants (Fig. 3a). The changes in ML-FA levels did not impact the ML-*p*CA release levels determined, as these levels were similar between all greenhouse-grown and field-grown plants (Fig. 3b). These data all align with the contention that *ccr1* mutants, with higher ML-FA levels and improved digestibility, are viable biofuel crops.

**Table 3:** Agronomic trait analysis of field-grown *Zmccr1* mutants

Trait	W22	*ccr1*(00732)	Significance
Plant height (cm)	173.7 ± 9.6^a^	187.2 ± 10.3	*
Ear height (cm)	79.8 ± 9.0	87.1 ± 8.4	*
Above-ear height (cm)	93.9 ± 7.7	100.1 ± 7.5	*
Node number	15 ± 0.5	16 ± 0.8	*
Below-ear node number	8 ± 0.6	8 ± 0.6	ns
Above-ear node number	7 ± 0.5	7 ± 0.6	*
Internode length (cm)	11.4 ± 0.7	11.9 ± 0.6	*
Above-ear internode length (cm)	13.4 ± 0.5	13.6 ± 0.8	ns
Below-ear internode length (cm)	9.7 ± 1.0	10.3 ± 0.7	*
Days to flower	71.3 ± 0.5	76.3 ± 1.5	*
Leaf length (cm)	44.9 ± 4.6	49.8 ± 3.8	*
Leaf width (cm)	9.5 ± 0.6	9.6 ± 0.8	ns
Stalk diameter (cm)	3.1 ± 0.2	3.1 ± 0.2	ns
Stover weight (gr)	73.7 ± 3.7	94.7 ± 9.1	*
Lodging (%)	3.106 ± 3.752	11.998 ± 13.672	ns
Seed weight (gr)	60.482 ± 8.006	50.328 ± 9.601	*

*ns*Non-significant

*Significance level of 0.05 (*t* test)

^a^Standard deviation

## Conclusions

Increasing the biosynthesis of monolignol ferulates, and thereby increasing the digestibility of the cell wall, appears to be regulated by at least one key factor. In maize, the amount of feruloyl-CoA substrate appears to be a limiting factor in the production of monolignol ferulates by a putative *Zm*FMT. The increased monolignol ferulate incorporation into lignins in *ccr1* mutant plants suggests that they may provide superior biofuels substrates, and even higher stover yields, and could have implications for viable methods by which to increase monolignol ferulate levels in other biofuel crops, such as trees or sorghum.

## Methods

### Plant growth conditions and field evaluation

F_3_ generation *CCR1* mutant UniformMu seed stocks UFMu-00732 (*Zmccr 1a*) and UFMu01379 (*Zmccr 1b*) harboring the Mu transposon mu1013391 in exon four of the *CCR1* gene (GRMZM2G131205, Chr1: 211567137.0.211573211, http://www.maizegdb.org) (Additional file 1: Fig. 1A) were obtained from the Maize Genetics Cooperation Stock Center (http://www.maizegdb.org/ordering/stock). Mutant plants grown from F_3_ seeds were identified by a PCR assay and were self-pollinated. Homozygous *CCR1* mutant progenies and wild type (W22 inbreds) were planted in a greenhouse (27 °C day, 24 °C night, 16 h day (light), 8 h dark).

*Arabidopsis thaliana cinnamoyl-CoA reductase1* (*ccr1* ^*irx4*^; AT1G15950) mutants were obtained from the Arabidopsis Biological Resource Center (ABRC) and were grown to senescence in a growth chamber (21 °C day, 16 h day (light), 8 h dark).

For maize digestibility assays and the measurement of lignin, monomer composition (by thioacidolysis), and monolignol conjugates (by DFRC), the third internode below the topmost ear-bearing node was harvested at physiological maturity (40 days after anthesis). Agronomic traits were measured on one-row plots in a randomized complete block design with six replications at the West Madison Research Station in Wisconsin from May to September, 2016. Plant height and ear height were measured from soil surface to the flag leaf node and to the top ear node, respectively. The average internode length was estimated by dividing height by node number. Days to flower were recorded when more than half of the plants within a plot shed pollen. The second top leaf was used for taking leaf measurements. Leaf length was measured from the tip of the leaf to the leaf blade, and leaf width was measured from the leaf mid-region. Stalk diameter was calculated based on the circumference of the second above-ground internode. Lodge percentage was estimated based on the portion of lodged plants within each plot. Ears and stover were collected two months after the flowering date and were kept in a drier at 55 °C for a week after which seed and stover weight were measured.

### PCR and gene expression

Genotyping for the Uniform Mu insertion mu1013391 found in seed stocks UFMu00732 and UFMu01379 in *Zmccr1* (GRMZM2G131205, Chr1: 211567137.0.211573211, http://www.maizegdb.org) was performed using primers indicative of either the Uniform Mu insertion (T1 forward 5′-AACGCCTCCATTTCGTCGAATCC-3′, R1 reverse 5′-GGTACTCGGGGAAGAGCTTGGC-3′, 375-bp PCR product) or the wild-type *CCR1* allele (F1 forward 5′-TACGACTTTGGTGGGTCGCTCG-3′ and the R1 reverse primer, 537-bp PCR product). Amplification conditions for the T1-R1 primer pair were as follows: 94 °C—3 min, one cycle; dissociation 94 °C—30 s, annealing 60 °C—30 s, extension 72 °C—20 s, 36 cycles total; 72 °C—10 min, one cycle; 4 °C–hold; conditions for the F1-R1 primer pair were similar except that the annealing temperature was 59 °C and the extension time was 30 s (Additional file 1: Fig. 1A, B). Sequenced PCR products confirmed that the mu1013391 was inserted into the GRMZM2G131205 locus, as reported by flanking sequence GenBank HQ140720.

For expression analysis, 500 ng of leaf total RNA was reverse-transcribed by MMLV RT (Promega) using oligo (dT)_18_ following the manufacturer’s instructions. *Zmccr1* expression level was determined by semi-quantitative PCR (real time-sqPCR) using 100 ng of leaf first-strand cDNA in a 20-µL reaction volume, and F2 forward 5′- TCCTCGCCAAGCTCTTCCCCGA-3′ and R2 reverse 5′-AAGAACGAACATGACGTTACAAGTCTTAGG-3′ primers that produced a 408-bp PCR product (Additional file 1: Fig. 1A, C). To ensure equal loading, *Zea mays* GAPDH (GRMZM2G180625) was used as a reference gene (forward 5′-GACCTCACTGTCAGAATCGAGAAGG-3′ and reverse 5′-CACTCGTTGTCGTACCAAGAGACG-3′ primers, 224-bp PCR product). Amplification conditions were 96 °C—3 min, one cycle; 96 °C—30 s, 55 °C—30 s, 72 °C—30 s, 28 cycles total; 72 °C—10 min, one cycle; 4 °C—hold. The PCR products were verified by sequencing.

### Chemical analysis

#### Maize tissue sample

All chemical assays were performed on the same homogenized maize rind tissue. The third internode below the topmost ear-bearing node was harvested from maize plants (*WT*; *ccr1* mutant) at physiological maturity (40 days after anthesis) and dried in an oven at 42 °C for a week. The rind was isolated and ground to a fine powder using a Retsch mill at 30 1/s frequency for 2–3 min. Stem tissues were solvent extracted [water (3 × 40 mL), 80% ethanol (3 × 40 mL) and acetone (1 × 40 mL)] and freeze-dried for 2 days. The resulting powder was then homogenized and distributed for analysis.

Mature stem material from Arabidopsis *ccr1* ^*irx4*^ mutants (pool of 10 plants) was isolated, ground, and solvent extracted, as described above.

#### Klason lignin analysis

Acid-insoluble lignin was determined using Klason lignin analysis of 150–200 mg extractive-free rind tissue, as previously described [15]. Acid-soluble lignin was determined by measuring the absorbance at 205 nm of the filtrate after isolation of acid-insoluble lignin and calculated using an extinction coefficient of 110 L/g cm.

#### Thioacidolysis

Thioacidolysis was performed at the Michigan State University’s Cell Wall Facility to determine lignin composition following the original procedure [16, 17]. Briefly, the thioacidolysis reagent (2.5% (v/v) boron trifluoride diethyletherate, 10% (v/v) ethanethiol in fresh dioxane) was spiked with 4,4′-ethylidenebisphenol (1 mg/mL in dioxane) as an internal standard. Thioacidolysis monomers were extracted after 4 h at 100 °C, then silylated with *N,O*-bis(trimethylsilyl)trifluoroacetamide and pyridine, and quantified by GC/MS (Agilent GC/MS, 6890 GC and 5975B MS) fitted with a Supelco SLB-5MS column (30 mm × 0.25 mm × 0.25 μm film) using synthetic thioacidolysis monomers as standards. The linear range and response factor (RF) for the synthetic monomers versus the internal standard (bisphenol E, ion 343) were as follows: for S, 25–300 µg, *r* ^2^ = 0.998, RF (ion 299) = 2.16; for G, 25–300 µg, *r* ^2^ = 0.998, RF (ion 269) = 2.15; and for H, 2.5–50 µg, *r* ^2^ = 0.998, RF (ion 239) = 2.11. Each line represents an average of five biological replicates composed of two technical replicates each. The error bars in the graphical results represent the standard deviation between the biological replicates. Statistical analysis was performed by paired *t* tests between *ccr1* mutants and corresponding wild-type plants with a significance level of 0.05.

#### Derivatization followed by reductive cleavage (DFRC) method for lignin-bound ML-FAs

The quantitation of lignin-bound *ML*-*FAs* was performed using the DFRC method that cleaves lignin β-ethers while retaining esters [13, 18–21], as recently described [3]. The same dry extract-free whole cell walls used for Klason lignin analysis (50 mg) were treated with a solution of acetyl bromide (AcBr) in acetic acid (20%, v/v) at 50 °C for 2.5 h in a 3 dram vial with a vacuum-sealed cap. The acetylated and benzyl-brominated lignin solution was dried under vacuum for 40 min at 50 °C. The dry film was treated with absolute ethanol (1 mL), which was then also removed on a SpeedVac concentrator at 50 °C for 15 min. The dry sample was then immediately dissolved in a mixture of 1,4-dioxane:acetic acid:water (5:4:1, by volume, 5 mL), and zinc nano-powder (150 mg) was added to the vial. The reaction was stirred for 16 h at room temperature and then quenched with saturated ammonium chloride. The quenched reaction was spiked with an internal standard mixture (deuterated monolignols and conjugates). The organics were extracted with DCM (3 × 15 mL) and the combined organic fractions were dried over sodium sulfate. The DCM was removed under vacuum, and the free hydroxyl groups were acetylated overnight using a mixture of acetic anhydride and pyridine (1:1, v/v). The excess acetic anhydride and pyridine were removed on a rotary evaporator, after which the crude product was loaded onto a Supelco Supelclean LC-SI SPE tube (Sigma-Aldrich) with the aid of ethyl acetate and hexane (1:1, ~1 mL). The purified product was then eluted using a mixture of ethyl acetate:hexane (1:1, 8 mL), and concentrated to dryness. The dry film was dissolved in DCM (1 mL) and injected into a Shimadzu GCMS-TQ8030 triple-quadrupole GC/MS/MS operating in multiple-reaction-monitoring (MRM) mode for quantitative analysis (using calibration curves derived from synthetic standards). Each line represents an average of five biological replicates composed of two technical replicates each for greenhouse-grown lines, and an average of two pools of ten plants for field-grown lines. The error bars represent the standard deviation between the biological replicates. Statistical analysis was performed by paired *t* tests between *ccr1* mutants and corresponding wild-type plants with a significance level of 0.05.

#### Digestibility analysis

Digestibility analysis to yield glucose and pentoses was performed as described in [22], using ~2 mg, accurately weighed, of mature rind tissue sample for each of three technical replicates. Each line represents an average of five biological replicates for greenhouse-grown lines, and an average of two pools of ten plants for field-grown lines. Statistical analysis was performed by paired *t* tests between *ccr1* mutants and corresponding wild-type plants with a significance level of 0.05.

## Additional file

**Additional file 1.** **Figure** 1. Molecular characterization of the mu1013391 insertion in *Zmccr1*. **A)** Scale diagram of the *ZmCCR1* locus GRMZM2G131205, Chr1: 211567137..211573211. Black and white boxes represent exons and untranslated regions, respectively, while lines indicate introns. Shown is the location of the Uniform Mu insertion mu1013391 (triangle) in seed lots UFMu00732 (*Zmccr 1a*) and UFMu01379 (*Zmccr 1b*) along with relative primer locations (arrows). **B)** Shown are agarose gel-electrophoresed PCR products indicating either the presence (primers T1 + R1, 375 bp) or absence (primers F1 + R1, 537 bp) of the mu1013391 insertion in either plants homozygous for the mu1013391 insertion (*Zmccr 1a* and *1b*) or wild-type W22. **(C)** Agarose gel-electrophoresed PCR products semi-quantitatively amplified from reverse-transcribed first-strand cDNA from plants either homozygous for the mu1013391 insertion (*Zmccr 1a* and *1b*) or WT B73 (primers F2 + R2, ~408 bp). Note that the *Zmccr 1a* and *1b* PCR products appear larger than that of WT B73 due to a 12-bp insertion. GAPDH was used as a loading control (224 bp). NT = no template.** Figure** 2. Raw DFRC data. A) GC-MS chromatograms of coniferyl dihydro-ferulate (G-DHFA) conjugates from wild-type and *ccr1* Maize plants, and the Arabidopsis *ccr1* mutant, *irx4*; the highlighted region the position of the *trans* isomer. The asterisks indicate the sinapyl dihydro-*p*-coumarate peaks (*cis* and *trans*) in the maize samples that also have this MRM transition, but elute at different retention times. B) The GC-MS chromatograms of sinapyl dihydro-ferulate (S-DHFA) conjugates from Maize and Arabidopsis samples. The first highlighted peak is *cis*-S-DHFA and the second is *trans*-S-DHFA. Note that the peaks shown are from the molecular ion minus ketene (CH_2_=C=O); this can come from the acetate on either end of the conjugate (as shown by the dotted bond).

## Abbreviations

FMT: FERULOYL-CoA MONOLIGNOL TRANSFERASE
CoA: coenzyme A
CCR: CINNAMOYL-CoA REDUCTASE
ccr: CCR-suppressed mutant
PMT: *p*-COUMAROYL-CoA MONOLIGNOL TRANSFERASE
*p*CAT: *p*-COUMAROYL-CoA ACYLTRANSFERASE
ML: monolignol
FA: ferulate
*p*CA: *p*-coumarate
H: 4-hydroxyphenyl
G: 3-methoxy-4-hydroxyphenyl
S: 3,5-dimethoxy-4-hydroxyphenyl
ML-FA: monolignol ferulate conjugate
ML-*p*CA: monolignol *p*-coumarate conjugate
NaOH: sodium hydroxide
*Zm*: *Zea mays*
WT: wild type
RNA: ribonucleic acid
RNAi: ribonucleic acid interference
cDNA: complementary deoxyribonucleic acid
PCR: polymerase chain reaction
RT: reverse transcriptase
sqPCR: semi-quantitative polymerase chain reaction
MMLV: Murine leukemia virus
GAPDH: glyceraldehyde 3-phosphate dehydrogenase
bp: base pair
v/v: volume to volume
h: hour
min: minute
GC: gas chromatography
MS: mass spectrometry
MRM: multiple reaction monitoring
RF: response factor
AcBr: acetyl bromide
DCM: dichloromethane
AIR: alcohol insoluble residue
DFRC: derivatization followed by reductive cleavage
AFEX: ammonia fiber expansion
DOE: United States of America Department of Energy
