# Supplementary material for: Suppression of CINNAMOYL-CoA REDUCTASE increases the level of monolignol ferulates incorporated into maize lignins
Source: Biotechnol Biofuels. 2017 May 2;10:109. doi: 10.1186/s13068-017-0793-1 (PMC5414125; doi:10.1186/s13068-017-0793-1)
Supplement: Supplementary file 1 — Additional file 1. Figure 1. Molecular characterization of the mu1013391 insertion in Zmccr1. A) Scale diagram of the ZmCCR1 locus GRMZM2G131205, Chr1: 211567137..211573211. Black and white boxes represent exons and untranslated regions, respectively, while lines indicate introns. Shown is the location of the Uniform Mu insertion mu1013391 (triangle) in seed lots UFMu00732 (Zmccr 1a) and UFMu01379 (Zmccr 1b) along with relative primer locations (arrows). B) Shown are agarose gel-electrophoresed PCR products indicating either the presence (primers T1 + R1, 375 bp) or absence (primers F1 + R1, 537 bp) of the mu1013391 insertion in either plants homozygous for the mu1013391 insertion (Zmccr 1a and 1b) or wild-type W22. (C) Agarose gel-electrophoresed PCR products semi-quantitatively amplified from reverse-transcribed first-strand cDNA from plants either homozygous for the mu1013391 insertion (Zmccr 1a and 1b) or WT B73 (primers F2 + R2, ~408 bp). Note that the Zmccr 1a and 1b PCR products appear larger than that of WT B73 due to a 12-bp insertion. GAPDH was used as a loading control (224 bp). NT = no template. Figure 2. Raw DFRC data. A) GC-MS chromatograms of coniferyl dihydro-ferulate (G-DHFA) conjugates from wild-type and ccr1 Maize plants, and the Arabidopsis ccr1 mutant, irx4; the highlighted region the position of the trans isomer. The asterisks indicate the sinapyl dihydro-p-coumarate peaks (cis and trans) in the maize samples that also have this MRM transition, but elute at different retention times. B) The GC-MS chromatograms of sinapyl dihydro-ferulate (S-DHFA) conjugates from Maize and Arabidopsis samples. The first highlighted peak is cis-S-DHFA and the second is trans-S-DHFA. Note that the peaks shown are from the molecular ion minus ketene (CH2=C=O); this can come from the acetate on either end of the conjugate (as shown by the dotted bond). [file 13068_2017_793_MOESM1_ESM.pdf]

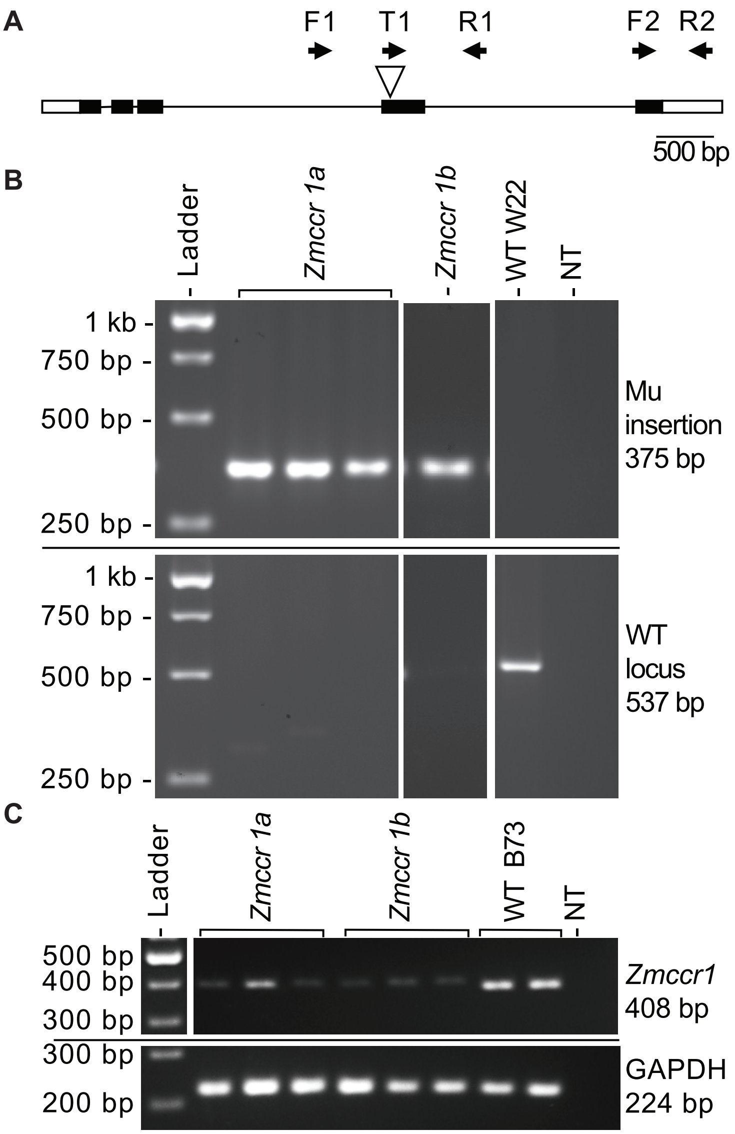

Supplemental Figure 1. Molecular characterization of the mu1013391 insertion in *Zmccr1*. A) Scale diagram of the *ZmCCR1* locus GRMZM2G131205, Chr1: 211567137..211573211. Black and white boxes represent exons and untranslated regions, respectively, while lines indicate introns. Shown is the location of the Uniform Mu insertion mu1013391 (triangle) in seed lots UFMu00732 (*Zmccr 1a*) and UFMu01379 (*Zmccr 1b*) along with relative primer locations (arrows). B) Shown are agarose gel-electrophoresed PCR products indicating either the presence (primers T1 + R1, 375 bp) or absence (primers F1 + R1, 537 bp) of the mu1013391 insertion in either plants homozygous for the mu1013391 insertion (*Zmccr 1a* and *1b*) or wild-type (WT) W22. C) Agarose gel-electrophoresed PCR products semi-quantitatively amplified from reverse-transcribed first strand cDNA from plants either homozygous for the mu1013391 insertion (*Zmccr 1a* and *1b*) or wild-type (WT) B73 (primers F2 + R2, ~408 bp). Note that the *Zmccr 1a* and *1b* PCR products appear larger than that of wild-type B73 due to a 12 bp insertion. GAPDH was used as a loading control (224 bp). NT = no template.

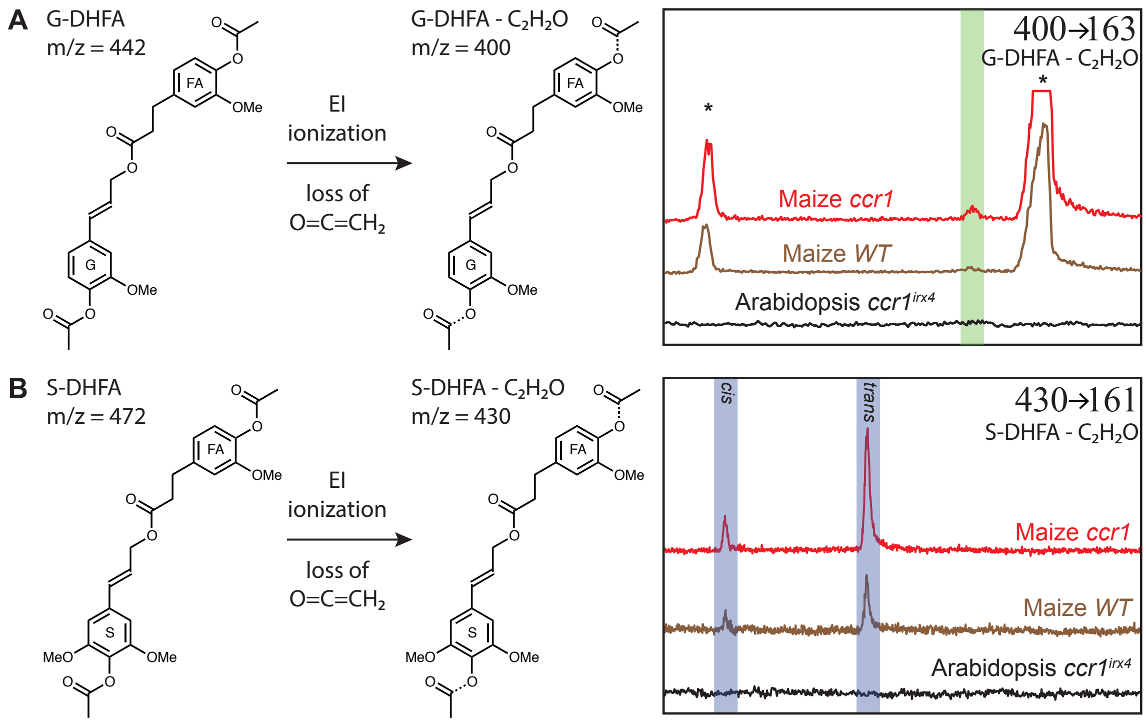

Supplemental Figure 2. Raw DFRC data A) GC-MS chromatograms of coniferyl dihydroferulate (G-DHFA) conjugates from Maize wild-type and *ccr1* Maize plants and the Arabidopsis *ccr1* mutant, *irx4*; the highlighted region shows the position of the *trans* isomer. The asterisks indicate the sinapyl dihydro-p-coumarate peaks (*cis* and *trans*) in the maize samples that also have this MRM transition, but elute at different retention times. B) The GC-MS chromatograms of sinapyl dihydroferulate (S-DH-FA) conjugates from Maize and Arabidopsis samples. The first highlighted peak is *cis*-S-DHFA and the second is *trans*-S-DHFA.
